# Patient Care Satisfaction and Emergency Room Utilization among Young Adult Colorectal Cancer Survivors during the SARS-CoV-2 Pandemic: Lessons Learned

**DOI:** 10.3390/jcm12020469

**Published:** 2023-01-06

**Authors:** Dalia Kagramanov, Kimberly A. Miller, Phuong Gallagher, David R. Freyer, Joel E. Milam, Heinz-Josef Lenz, Afsaneh Barzi

**Affiliations:** 1 Department of Population and Public Health Sciences, Keck School of Medicine, University of Southern California, Los Angeles, CA 90033, USA; 2 Department of Dermatology, Keck School of Medicine of the University of Southern California, Los Angeles, CA 90033, USA; 3 The Colon Club, Pasadena, CA 91105, USA; 4 Cancer and Blood Disease Institute, Children’s Hospital Los Angeles, Los Angeles, CA 90027, USA; 5 Department of Pediatrics, Keck School of Medicine of the University of Southern California, Los Angeles, CA 90033, USA; 6 Department of Medicine, Keck School of Medicine of the University of Southern California, Los Angeles, CA 90033, USA; 7 USC Norris Comprehensive Cancer Center, Los Angeles, CA 90033, USA; 8 Department of Epidemiology and Biostatistics, Chao Family, Comprehensive Cancer Center, School of Medicine, University of California, Irvine, CA 92868, USA; 9 Department of Oncology, University of Southern California, Norris Comprehensive Cancer Center, Los Angeles, CA 90033, USA; 10 Department of Medical Oncology and Therapeutics Research, City of Hope National Medical Center, Duarte, CA 91010, USA

**Keywords:** colorectal cancer survivor, emergency utilization, SARS-CoV-2, care satisfaction, pandemic

## Abstract

Introduction: Survivors of colorectal cancer (CRC) are at risk for late effects of therapy and recurrence of cancer. With recurrence rates ranging between 30–40%, follow-up care is needed for both early detection and management of late effects. Cancer care delivery for CRC patients was significantly disrupted by the SARS-CoV-2 pandemic, with decreases of 40% in such services in the United States between April 2020 and 2019. Survivors were left with fewer options for care, potentially causing increases in emergency room (ER) utilization. Methods: This cross-sectional study examined the patterns of ER utilization during the SARS-CoV-2 pandemic among young adult CRC survivors and assessed the relationship between self-reported care satisfaction and ER use. Eligible participants were colon or rectal cancer survivors diagnosed between 18–39 years of age, 6–36 months from diagnosis/relapse, English speaking and residing in the United States. Multivariable logistic regression assessed the association between patient care satisfaction and ER utilization, adjusting for pandemic factors. Covariates were chosen by significance of *p* < 0.1 at the univariate level and perceived clinical significance. Results: The overall sample (N = 196) had mean age (SD) 32.1 (4.5); 59% were male. Tumor location was colon or rectal in 42% and 57%, respectively, and the majority (56%) were diagnosed with stage 2 disease; 42.6% reported relapsed disease, and 20% had an ostomy. Most survivors (72.5%) had between 1–4 visits to an ER in the last 12 months and were categorized as normal utilizers. Approximately 24.7% of the sample had greater than 4 visits to the ER in the last 12 months and were categorized as super-utilizers. CRC survivors that reported a delay in their follow-up care as a result of the pandemic were two times (OR: 2.05, 95% CI 0.99, 4.24) more likely to be super-utilizers of the ER. Higher self-reported satisfaction with care was associated with a 13.7% lower likelihood of being a super-utilizer (OR: 0.86, 95%CI: −0.68, 1.09). Conclusions: This study found strong associations between delays in care, self-reported care satisfaction, and being a super-utilizer of the ER during the pandemic among young adult CRC survivors off treatment. Increasing patient satisfaction and minimizing care interruptions amongst this vulnerable population may aid in mitigating over-utilization in the ER during an ongoing pandemic.

## 1. Introduction

Colorectal cancer (CRC) is the second cause of cancer-related death and the third most common cancer in the United States [1]. Upon diagnosis, treatment plans are often multimodal, involving a combination of chemotherapy, radiation, and surgery. With advancements in early detection and improved treatment modalities, nearly two-thirds of CRC survivors are living more than 5 years post diagnosis [2]. However, survivors of this malignancy are at risk for late effects of therapy, as well as recurrence of cancer. Side effects may include both physical ailments and/or psychosocial complications [3]. Additionally, overall recurrence rates for colorectal cancer remain high, ranging between 30–40% among survivors [2].

The incidence of colorectal cancer has continued to rise among young adults aged 18–39 years [4]. These are individuals who face unique challenges navigating cancer care during the transformative stages of young adulthood which commonly include changes in residence, career development, starting a family, etc. Post-treatment concerns characteristic of young adulthood include reproductive health, genetics, social impacts, and future employment [5].

Consistent follow-up care is critical for identifying late-effects and recurrence [6], thereby increasing one’s chances for long-term survival. Follow up care is also a venue for assisting survivors to develop coping strategies and access resources for ongoing issues [7]. Common survivorship care models where follow-up care is conducted include multidisciplinary care, characterized by a dedicated team of healthcare professionals who provide a range of health services to survivors, and shared care models which include collaborative care between the oncologist and primary care provider (PCP) [8]. Considering the high rates of recurrence among CRC survivors, regular cancer-directed screening and physical examinations via any survivorship care model are of high importance. Both the National Comprehensive Cancer Network (NCCN) and the American Society of Clinical Oncology (ASCO) have developed survivorship care guidelines for clinicians to utilize when working with cancer survivors to prevent and support late-effects of treatment. However, attendance rates to survivorship care models overall among young adult survivors remain low [9]. Further, the literature on CRC has shown that higher care density, the extent to which a patient’s providers share patients with one another, and lower care fragmentation are associated with a reduced likelihood of hospitalization and emergency room visits [10].

The SARS-CoV-2 pandemic had a drastic impact on health care globally, including cancer treatment care and survivorship care. CRC-related care delivery was significantly disrupted with decreases of up to 40% in CRC services in the United States between April 2020 and April 2019 [11]. As a result, survivors may have been left with few options for cancer-related care. With the feeling of uncertainty that accompanied the global pandemic, disruptions in available survivor care services may have played a role in potentially driving emergency room utilization higher in this population. Additionally, delays in receiving care as a result of the pandemic may have also impacted patients’ care satisfaction with cancer specialists or general care providers, further impacting the utilization patterns in this vulnerable population with potential long-term consequences. SARS-CoV-2 created an unprecedented disruption in care, social norms, and health care expectations, including the impactful changes in the experiences of cancer survivors. The impact of disruptions in care during SARS-CoV-2 (especially the earlier period) may be viewed as a social experiment to understand the interaction between perception of access to care by patients and utilization of services. The lessons of this social experiment can pave the way to design more robust systems and to equip future patients and survivors with tools to address their unmet needs. Although the effects of the COVID-19 pandemic were unprecedented, delays in care can happen for a variety of reasons and are likely to occur in the future. Deeper understanding of the needs of CRC survivors during these times will help to reduce negative impacts.

We set to explore the patterns of emergency room utilization among young adult CRC survivors in the United States during SARS-CoV-2 pandemic. Using survey data from a national society for young adult survivors of colorectal cancer, we explored the patterns of ER utilization during this time and assessed the relationship between self-reported satisfaction with care and emergency-care use. We hypothesized that lower self-reported satisfaction measures would be associated with greater emergency-room utilization.

## 2. Materials and Methods

This cross-sectional study was conducted using an online survey administered on the Facebook page of a national Colorectal Cancer advocacy group between 31 August and 3 September 2020. Participants were eligible if they were colon or rectal cancer survivors, aged 18–39 at time of diagnosis, between 6–36 months from diagnosis or relapse, English speaking and based in the United States. Study procedures have been detailed elsewhere [4]. An electronic gift card valued at USD20 was provided to participants who completed the survey. The study was approved by the University of Southern California Institutional Review Board (IRB).

### 2.1. Data Verification

The data cleaning process aimed to ensure validity and reduce fraudulent responses inherent within social media recruitment. Participants were asked questions regarding eligibility at the start of the survey to eliminate automated software or “bots.” Additionally, duplicate email use was prohibited. This was monitored by removing respondents whose survey completion time was less than five minutes, given an average completion time of 17-min. Lastly, respondent data was removed if reporting included “highly improbable” medical treatment patterns as reviewed by a medical oncologist [4].

### 2.2. Variables

Participants were surveyed regarding general demographic information, cancer-related treatment data, five self-reported satisfaction with care questions, scaled 0–10, and the number of emergency room visits in the last 12 months. The collection of survey questions was created from previously validated scales or measures widely used in cancer research. For example, CRC survivors self-reported gender, race/ethnicity, age, stage at diagnosis, whether they had experienced relapsed disease and the year of most recent relapse. Questions on care satisfaction were based on the Consumer Assessment of Healthcare Providers and Systems (CAHPS) patient experience survey (https://www.ahrq.gov/cahps/surveys-guidance/index.html URL accessed on 1 August 2020) [12]. Additionally, survivors were asked about a number of pandemic-related questions such as delays in access to care, financial impacts, psychological and emotional distress, and job loss [13]. These were based on questions from The Pandemic Stress Index [14]. The pandemic-related variables were then also used as co-factors in the statistical modelling process. The full scope of the survey was also pilot tested and reviewed by a patient advocate (P.G.) to ensure the questions used were acceptable and comprehensible to the target group of young adult CRC survivors.

### 2.3. Statistical Analysis

Frequencies and percentages were calculated for sample demographics, emergency care utilization, and self-reported satisfaction of care measures. A multivariate logistic regression was conducted on the overall sample to assess the association between patient satisfaction and emergency room utilization, adjusting for the influence of the COVID-19 global pandemic. Covariates for this analysis were chosen based on a significance of *p* < 0.05 at the univariate level, as well as general clinical significance. The demographic/clinical characteristic covariates included in the multivariable model were sex, race/ethnicity, and age at diagnosis. Treatment intensity was assessed as a potential covariate in the relationship between prior cancer therapy and the outcomes of ER utilization and patient care satisfaction. Treatment intensity was calculated as the sum of the self-reported treatment modalities received (chemotherapy, radiation, surgery, and/or immunotherapy) and scored on a scale from 0 (defined as no therapy received) to 4 (defined as receiving all four modalities). This covariate was not statistically significant at the univariate level and was therefore not included in the final multivariable model.

Based on similar prior literature on emergency room utilization, the outcome of emergency room utilization was dichotomized. Survivors who visited an emergency room greater than 4 times in the last 12 months were termed “super-utilizers” while survivors who visited an emergency room less than or equal to 4 times in the last 12 months were considered “normal utilizers” [15]. All statistical analyses were performed using SAS (Version 9.4).

## 3. Results

A total of 371 survey responses were received, of which 196 (53%) were retained after screening eligibility criteria and removing responses that were identified as potentially fraudulent based on our previous algorithm. Sample characteristics are presented in Table 1. Overall mean age (SD) was 32.1 years (4.5), and 116 survivors (59%) were male. Diagnosis tumor location was colon or rectal in 39% and 61%, respectively, and the majority (56%) were diagnosed with stage 2 disease. Relapsed disease was reported by 58% of respondents, and 30% had an ostomy. Lastly, the majority of respondents were non-Latino white (79%).

**Figure 1:**
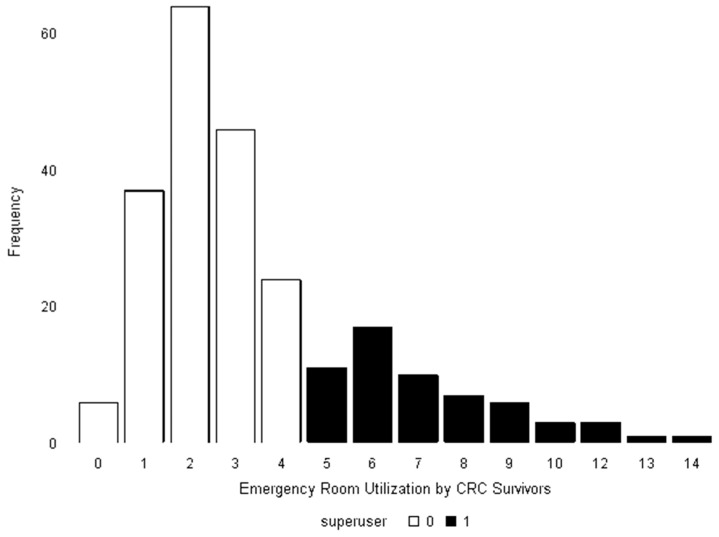
Distribution of “super-utilizers” of the emergency room among adolescent and young adult colorectal cancer survivors.

Approximately one quarter of the sample were super-utilizers of the emergency room (24.7%) (Figure 1). The majority of survivors (72.5%) had between 1–4 visits to an emergency room in the last 12 months.

Participants who had experienced a delay in their cancer care as a result of the pandemic were two times (OR: 2.05, 95% CI 0.99, 4.24) more likely to be super-utilizers of the emergency room. Additionally, those that experienced a delay in general care as a result of the pandemic were 92% more likely to be super-utilizers (OR: 1.92, 95% CI: 0.95, 3.86). However, this result was marginally significant at the *p* < 0.05 level. Survivors that had a higher self-reported care satisfaction rating for their primary provider were 23.5% (OR: 0.76, 95%CI: −0.60, 0.97) less likely to be super-utilizers of the emergency room. Similarly, higher self-reported satisfaction with overall care was associated with a 13.7% (OR: 0.86, 95%CI: −0.68, 1.09) less likely to be a super-utilizer. However, this result was not statistically significant. The described results are presented in Table 2.

## 4. Discussion

The patient care experience is an important aspect of health care quality and is associated with health care utilization and health outcomes. The results found in this study indicate that higher patient satisfaction with care was associated with lower use of the emergency room, which may be a result of perceived or actual changes in one’s access to care, as well as their experienced care. For example, clinic closures, delays in getting an appointment, or long wait times during scheduled appointments may have resulted in a perceived negative health care experience. Survivors as a result may be more likely to choose care from the emergency room for health-related concerns if they are unable or unwilling (due to poor experience) to access care elsewhere. Delays in care with one’ regular cancer care provider because of the pandemic may play a role in influencing a survivor’s future health behaviors. While the results of the cross-sectional study only look at one point in time, the highlighted associations shed light on areas for further study.

Emergency room utilization is a subject of interest in cancer care [16], with ongoing quality measures in development to reduce unnecessary use of this valuable resource [17]. The issue of higher use of ER due to lack of timely and proper access to outpatient services has been reported before [18], however, population-based research access has predominantly viewed and measured this though the channel of health insurance [19]. On the individual level, satisfaction with care can be a major determinant of perceived access to care. Therefore, poor satisfaction with care or sudden changes in an ongoing relationship with the care provider are barriers for access to appropriate care and thus increased use of ER.

In support of our findings, research on the general population has shown that higher patient satisfaction is associated with less emergency department use^7^. More so, previous literature also presented differences in care satisfaction and delays in care among Hispanic communities in the United States. Particularly, a study done in 2012 showed that non-Hispanic Black patient experience in the Los Angeles County may have an even greater impact on disease outcomes as a result of worse patient experiences with care being strongly associated with patient reports of discrimination [20]. This can further negatively impact health care utilization, driving individuals to seek care only in urgent cases through the emergency room. Due to the lack of diversity in the sample of this study, it is possible to have missed capturing even greater associations between self-reported care satisfaction and emergency room utilization among different race/ethnicity groups. Future research would benefit from obtaining data on a more diverse sample and stratifying analyses outcome measures by race/ethnicity groups.

Young adult colorectal cancer survivors commonly experience delays in care, financial hardship, and a reduced quality of life [15]. These components were further exacerbated for this vulnerable population during the SARS-CoV-2 pandemic when access to healthcare drastically changed and life for most was put on pause. With the knowledge that in-person care was greatly disrupted as a result of the pandemic [11], it is important to gain insight on the barriers and facilitators of this population’s health care utilization during such global events to aid in preparation of future care disruptions. This study found moderate to high associations between delays in care as a result of the SARS-CoV-2 pandemic and emergency room utilization, as well as self-reported satisfaction measures and emergency room utilization. It is possible that increased emergency care use by this population may be indicative of increased late effect symptomology during the pandemic, as well as a lack of obtaining recommended survivor care screening and physical assessments. Such changes in health behavior can have negative consequences on the health status of young adult CRC survivors.

The insight of our results provides valuable information on the potential drivers of this population’s health care patterns during the current SARS-CoV-2 pandemic. It is clear that CRC cancer care was disrupted in some form for this population of at-risk survivors, and it is important to recognize that changes in their ‘typical’ survivor-focused care can have great implications for their long-term outcomes. Added knowledge in the field can help to inform leaders on how to best support this vulnerable group in future health care disruptions. As a future direction, added exploration into the reasons for and nature of each emergency room visit will be beneficial towards understanding how to best care for these patients.

Some limitations of the interpretation of this study include the limited ability to infer causality based on the cross-sectional design, as well as the self-reported data being subject to bias. Despite rigorous attempts to reduce fraudulent responses, social media sampling prevented full verification of respondents’ patient status. Moreover, a social media sample may not be representative of the overall patient population as respondents were connected to an online resource and may represent a more motivated sample. Use of a social media survey also limits clinical verification of disease status. Lastly, considering the survey was conducted during the time of an unexpected pandemic, we do not have information on pre-pandemic ER usage patterns in this group, and are therefore unable to draw comparisons. Further information on methodological limitations is described in more detail in the parent study [4].

## 5. Conclusions

This study found strong associations between delays in care, self-reported care satisfaction, and emergency room utilization during the SARS-CoV-2 pandemic. The identification of such relationships adds valuable insight to the barriers and facilitators of care utilization during periods of extreme health care disruption. Importantly, survivors opting for emergency room use as opposed to regular follow-up care from their specialist or general care provider may be at risk for long-term consequences. Knowledge of these health behavior changes can help health care professionals recognize the impact of their individual approach and interactions on patient choice and facilitate interventions to improve such interactions. Undoubtedly, this learning can better prepare us for future global events, aiming to minimize cancer survivor impacts. Future research should aim to better characterize the relationship between patient satisfaction and with care and emergency room use in this at-risk population, as well as examine other common survivor populations, such as breast cancer survivors in the United States.

## Figures and Tables

**Table 1:** Characteristic of the sample overall and by age category *n* = 196.

	Current Age	
	20–29 (*n* = 56)	30–42 (*n* = 140)
**Sex**		
Male	33 (61.1)	83 (59.3)
Female	21 (38.9)	57 (40.7)
**Race/Ethnicity**		
Hispanic/Latino	8 (14.5)	12 (8.7)
Non-Hispanic White	41 (74.5)	112 (81.2)
Black/African American	3 (5.5)	10 (7.3)
Asian/Pacific Islander/Other	3 (5.5)	4 (2.8)
**Region**		
Midwest	6 (10.9)	31 (22.1)
Northeast	8 (14.6)	20 (14.3)
South	27 (49.1)	43 (30.7)
West	14 (25.4)	46 (32.9)
**Income Per Year**		
<USD35,000	14 (25.0)	18 (12.9)
USD35,000–USD74,999	26 (46.4)	92 (65.7)
USD75,000–USD149,999	15 (26.8)	28 (20.0)
>USD150,000	1 (1.8)	2 (1.4)
**Cancer Type**		
Colon	23 (42.6)	52 (38.0)
Rectal	31 (57.4)	85 (62.0)
**Stage At Diagnosis**		
Stage 1	18 (32.1)	25 (18.0)
Stage 2	23 (41.1)	87 (62.6)
Stage 3	13 (23.2)	23 (16.5)
Stage 4	2 (3.6)	4 (2.9)
**Relapse**		
Yes	23 (42.6)	89 (63.6)
**Ostomy**		
Yes	11 (20.0)	46 (33.6)

**Table 2:** Multivariable regression of ER super-utilization (>4 visits) and patient care satisfaction.

	OR	SE	95% CI	*ρ*
Gender				
Female	1.26	0.36	0.61, 2.57	0.53
Male (ref)				
Age at diagnosis	0.96	0.03	0.90, 1.02	0.19
Race/Ethnicity				
Hispanic/Latino	1.67	0.56	0.55, 5.02	0.36
Black/African American	0.65	0.69	0.17, 2.51	0.53
Asian/Pacific Islander/Other	0.47	1.25	0.04, 5.45	0.55
Non-Hispanic White (ref)				
Overall Healthcare Satisfaction Rating	0.86	0.12	0.68, 1.09	0.22
Primary Healthcare Provider Satisfaction Rating	0.77	0.12	0.60, 0.97	0.03
Specialist Healthcare Satisfaction Rating	1.00	0.11	0.81, 1.24	0.98
General Delays in Care (Past 12 months)	0.64	0.49	0.24, 1.69	0.37
Delays in Cancer Care Due to Pandemic	2.05	0.37	0.99, 4.24	0.05
Delays in General Care Due to Pandemic	1.92	0.36	0.95, 3.86	0.07

## Data Availability

The data presented in this study are available on request as a limited use dataset from the corresponding author. The data are not publicly available due to privacy considerations for participants.
